# Using Machine Learning and the National Health and Nutrition Examination Survey to Classify Individuals With Hearing Loss

**DOI:** 10.3389/fdgth.2021.723533

**Published:** 2021-08-18

**Authors:** Gregory M. Ellis, Pamela E. Souza

**Affiliations:** ^1^Department of Communication Sciences and Disorders, Northwestern University, Evanston, IL, United States; ^2^Knowles Hearing Center, Evanston, IL, United States

**Keywords:** audiology, remote audiology, machine learning, CDC, NHANES, centers for disease control and prevention, national health and nutrition examination survey

## Abstract

Even before the COVID-19 pandemic, there was mounting interest in remote testing solutions for audiology. The ultimate goal of such work was to improve access to hearing healthcare for individuals that might be unable or reluctant to seek audiological help in a clinic. In 2015, Diane Van Tasell patented a method for measuring an audiogram when the precise signal level was unknown (patent US 8,968,209 B2). In this method, the slope between pure-tone thresholds measured at 2 and 4 kHz is calculated and combined with questionnaire information in order to reconstruct the most likely audiograms from a database of options. An approach like the Van Tasell method is desirable because it is quick and feasible to do in a patient's home where exact stimulus levels are unknown. The goal of the present study was to use machine learning to assess the effectiveness of such audiogram-estimation methods. The National Health and Nutrition Examination Survey (NHANES), a database of audiologic and demographic information, was used to train and test several machine learning algorithms. Overall, 9,256 cases were analyzed. Audiometric data were classified using the Wisconsin Age-Related Hearing Impairment Classification Scale (WARHICS), a method that places hearing loss into one of eight categories. Of the algorithms tested, a random forest machine learning algorithm provided the best fit with only a few variables: the slope between 2 and 4 kHz; gender; age; military experience; and self-reported hearing ability. Using this method, 54.79% of the individuals were correctly classified, 34.40% were predicted to have a milder loss than measured, and 10.82% were predicted to have a more severe loss than measured. Although accuracy was low, it is unlikely audibility would be severely affected if classifications were used to apply gains. Based on audibility calculations, underamplification still provided sufficient gain to achieve ~95% correct (Speech Intelligibility Index ≥ 0.45) for sentence materials for 88% of individuals. Fewer than 1% of individuals were overamplified by 10 dB for any audiometric frequency. Given these results, this method presents a promising direction toward remote assessment; however, further refinement is needed before use in clinical fittings.

## Introduction

Several factors have been pushing audiologists toward telehealth, the most obvious of which is the COVID-19 pandemic. The pandemic closed the physical doors of audiology clinics around the world, requiring healthcare professionals to come up with alternatives to traditional in-person clinical approaches. Regardless of the pandemic, a shift to telehealth is necessary to reach underserved communities and individuals far away from audiology clinics.

One way to provide more convenient, accessible care for patients is to have them complete hearing tests in their own home. Testing hearing in the home is not a new concept. Computer-based or cellular phone-based hearing screenings (i.e., evaluating whether the participant can hear a preset level, and referring for further testing if they cannot) have been used successfully [e.g., (1–3)]. However, it is still more difficult to estimate hearing thresholds outside of an audiology testing center. Some at-home tests rely on a fairly traditional approach to audiometric testing, examining thresholds at octave frequencies between 250 and 8,000 Hz by providing a calibrated tablet and headphones. One such test, the Home Hearing Test, has been shown to produce reliable results in the home (4, 5). For a more thorough review of automated and in-home audiometric testing, please see Pragt et al. (6).

It is difficult to devise at-home hearing testing when the patient uses their own home computer or cell phone with earphones because that equipment will produce unknown presentation levels [for recent review of such approaches, see (7)]. A method for determining a patient's audiogram with limited audiological information was patented by Diane Van Tasell in 2015 (patent US 8,968,209 B2). In this method, pure-tone thresholds are measured at 2 kHz and 4 kHz. Rather than attempting to measure precise hearing thresholds at those frequencies, the slope between 2 and 4 kHz is calculated and combined with questionnaire information. Together, these data are used to reconstruct the most likely audiogram for that listener from a database of options. The method was intended to overcome the limitations of presenting accurate signal levels when using uncalibrated equipment. An approach like the Van Tasell method is desirable because it is relatively quick (only two thresholds in each ear are measured) and feasible to do in a patient's home on uncalibrated equipment where the exact levels of presented stimuli are unknown.

A similar in-home test would also be useful for experimental procedures. A large, diverse pool of subjects can be recruited and tested quickly by using remote testing. If the population of interest for a study is people with hearing impairment, it may be important to apply gain to the stimuli being tested. In this case, an estimate of the participant's hearing loss is necessary. Because a precise threshold cannot be guaranteed to be measured in the home for the reasons listed above, a remote testing solution that does not rely on precise threshold measurements is desirable.

Put plainly, the problem that needs to be solved is this: how can a person's audiometric thresholds be accurately predicted with limited information? Machine learning excels when using a set of features (variables) to categorize an unseen case. In order to do this, a machine learning algorithm is trained on a set of sample data, then it is asked to categorize a set of test data. By way of example, suppose a machine learning algorithm were trained to categorize objects as either an animal, a plant, or a mineral based on the object's features (e.g., shape, color, and size). If the algorithm was asked to categorize a strawberry, it would use the features it was trained on to make its best guess. Then the algorithm would—hopefully—correctly categorize the strawberry as a plant. The accuracy of any given machine learning algorithm is dependent on the particular cases it receives when it is being trained and how generally the algorithm is able to apply what it “learned” during the training phase. A large, diverse dataset tends to provide strong fits for a machine learning approach.

Fortunately, a large, diverse dataset of audiologic information exists in the public domain: the National Health and Nutrition Examination Survey (NHANES). NHANES is a complex survey that is collected biennially in the United States. Each survey cycle examines roughly 10,000 individuals from the United States civilian non-institutionalized population. Participants in the survey are given questionnaires, some are interviewed, and some receive medical examinations including audiometric tests. The NHANES database provides a rich source of pure tone audiometric and demographic data from individuals in the United States.

Audiometric data were categorized in order to facilitate most machine learning approaches (8). There are two major ways to categorize hearing losses that the authors are aware of today: the Wisconsin Age-Related Hearing Impairment Classification Scale (WARHICS) (9) and the IEC 60118-15 standard audiograms (10). Because the IEC standard audiograms are based on data from Stockholm (10) and the WARHICS classes were based on data collected in the United States (9), the WARHICS classes were used in the present study.

The goal of the present study was to determine how accurately a machine learning algorithm can predict a person's audiometric configuration given limited information about that person's demographics, hearing loss, and self-reported difficulty hearing. An additional goal was to apply this approach in a hypothetical speech test remotely administered, and to quantify the degree to which mismatches between the observed and predicted audiometric configurations would affect speech intelligibility. Three machine learning algorithms were trained using the following features: age, gender, previous military experience, the slope between 2,000 and 4,000 Hz pure tone thresholds, and self-reported amount of hearing difficulty.

## Materials and Methods

### Procedure

All data preprocessing and analysis was done in R (11) using the lattice (12), caret (13), Metrics (14), and tidyverse (15) packages.

Data were downloaded from the National Health and Nutrition Examination Survey (NHANES) database (https://www.cdc.gov/nchs/nhanes/index.htm). NHANES is a complex survey that studies the United States civilian non-institutionalized population. As a part of this survey, participants in most survey cycles receive audiometric evaluations. The large sample size and diverse population make NHANES an excellent dataset for examining audiometric patterns within the population surveyed. Because pure-tone thresholds were necessary for the present analysis, sample sets that did not include audiometric measurements were excluded. The sample sets that included audiometric data are those from 1999–2012 and 2015–2016. This span of years resulted in 71,963 cases.

Because of the complex survey design, special care needs to be taken when merging several datasets. These datasets were merged following the procedures outlined on the NHANES website in order to preserve sample weights. Sample weights are an important part of a complex survey, as they account for factors that make the selected sample more representative of the targeted population. Sample weights in the NHANES database take into account three major components. First, the sample weights account for the probability that a particular individual was selected to participate in the survey. Second, adjustments are made for non-response rates. Third, adjustments are made to account for oversampling of particular genders, age groups, and ethnic backgrounds.

It is also important to choose the appropriate set of weights. According to the NHANES site, a researcher must choose the weight that includes the smallest possible subpopulation that includes all of the variables of interest. The cases with audiometric data are the smallest subpopulation in the present study and the audiometric data were collected in the mobile exam center (MEC). Therefore, the MEC weights were used for the present study. Eight NHANES cycles were combined for this dataset. Based on the guidelines laid out in the NHANES tutorials, a combined weight was created by multiplying the weights from 1999–2002 by 0.25 and the weights for all other years by 0.125. These new weights were saved and used in analysis.

The survey questions asked of participants also changed over the years. The question of “General condition of hearing” (Which statement best describes your hearing (without a hearing aid)?) had six possible answers from 1999–2004 (AUQ130), eight possible answers from 2005–2010 (AUQ131), and was given a new designation starting in 2011 (AUQ054). The data needed to be adjusted for these changes. From 1999–2004, participants could answer: “Good,” “Little trouble,” “Lot of trouble,” “Deaf,” “Don't know,” or by refusing to answer the question. Starting in 2005, participants were given two new answers to the question: “Excellent,” and “Moderate hearing trouble.” When the data were merged, the class of answer from 1999–2004 was unchanged, though it should be noted that some number of participants that responded “Good” from 1999–2004 may have chosen “Excellent” if it were an option for them. A similar argument applies to the “Moderate hearing trouble” response added in 2005. For ease of reading, all three versions of this question (AUQ130, AUQ131, and AUQ054) will be referred to as “the question regarding hearing condition.”

**Figure 1:**
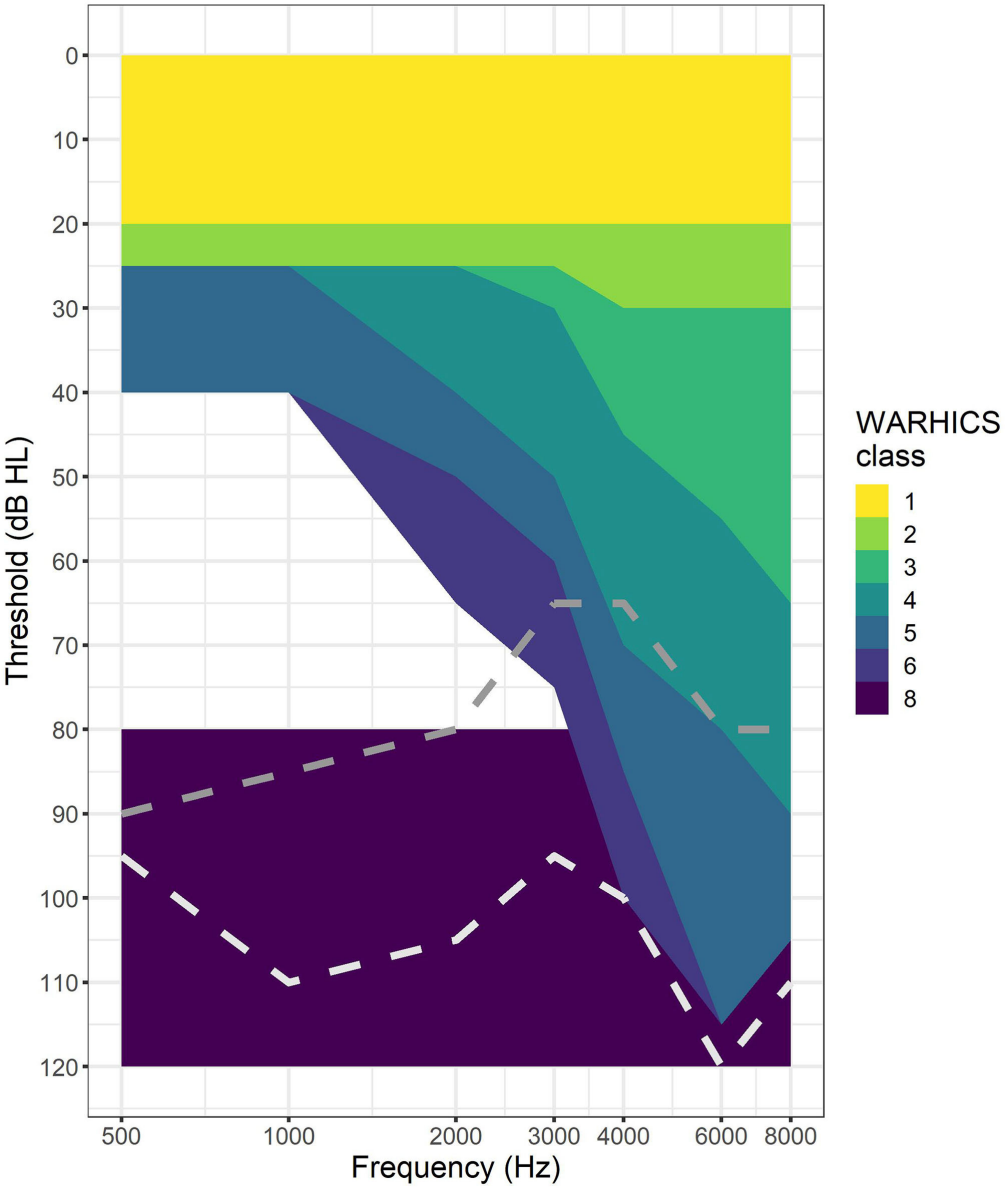
WARHICS classes plotted on an audiogram. Shaded regions represent regions in which an individual's audio must fall to be classified as that WARHICS class. Gray dotted line is an example from WARHICS class 7 (not 1–6 and at least one threshold < = 80 dB). White dotted line is an example from WARCHIS class 8 (all thresholds >= 80 dB).

Next, data were cleaned to ensure all cases had the following data: audiological thresholds for both ears at audiometric frequencies between 0.5 and 8 kHz, military experience, age, gender, and a response to the question regarding hearing condition. Of those 72,509 cases, 62,087 cases (85.6%) did not have audiological data because they did not participate in the MEC portion of their NHANES cycle. In addition to those missing audiological data, 1,164 cases (1.6%) were missing military status data. Two cases were missing answers to the question regarding hearing condition. All of these cases were dropped from the analysis resulting in 9,256 individuals with complete data for the variables listed above (12.8% of the original sample). Wisconsin Age-Related Hearing Impairment Classification Scale (WARHICS) classes were calculated for each ear for each person and saved as a separate variable for the two ears (WARHICS left and WARHICS right). The WARHICS subcategories were not included in this analysis because the subclasses were subsumed by the major classes in a previous study (9), and because the main classes were sufficient for the goals of the present study. See Figure 1 for a visualization of the WARHICS classes as they would appear on an audiogram.

### Demographics

**Figure 2:**
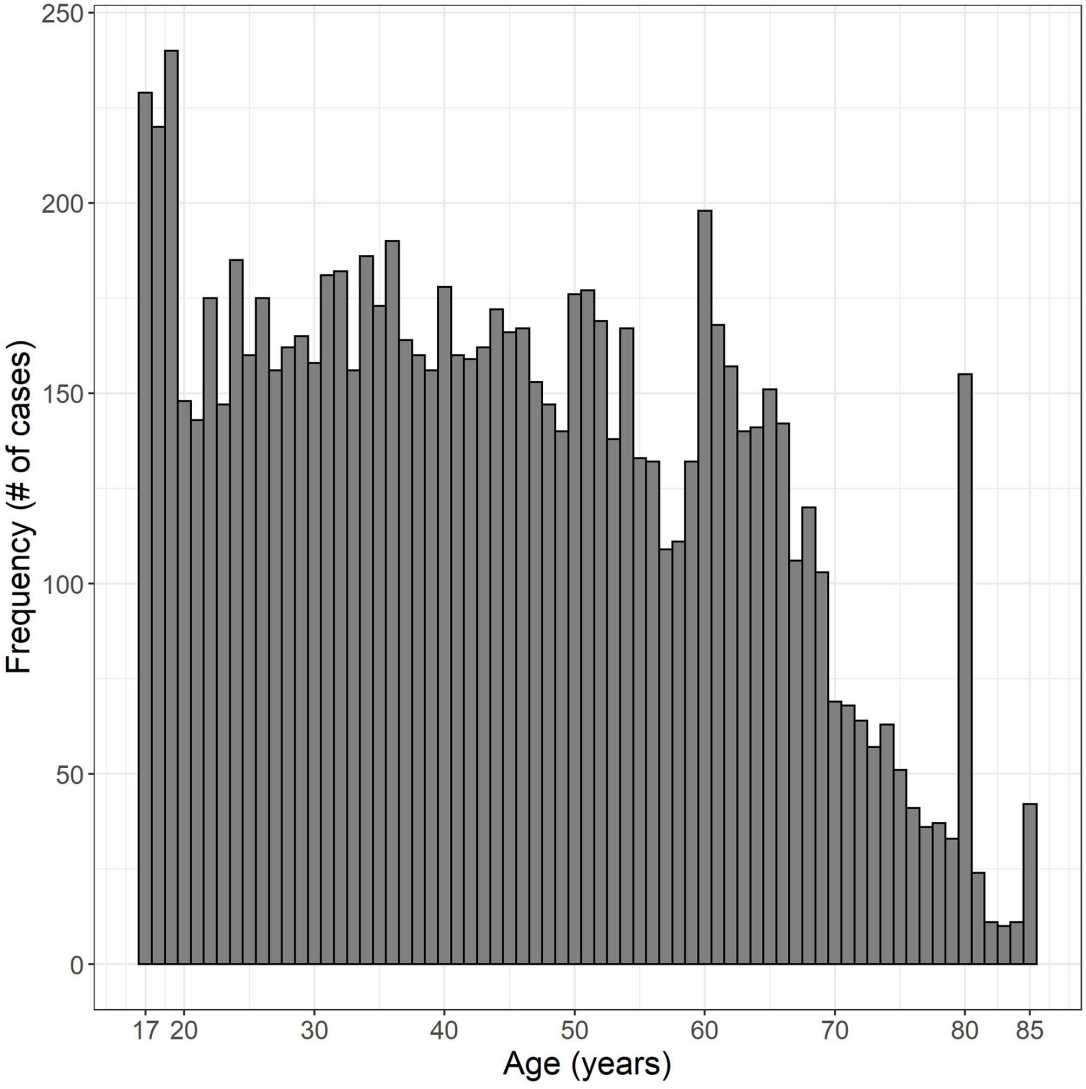
Histogram of ages examined. Data were obtained from the National Health and Nutrition Examination survey.

Of the 9,256 valid cases from 1999–2012 and 2015–2016, 4,156 cases (44.9%) were male and 5,100 cases (55.1%) were female. Eight hundred seventy-seven cases had military experience (9.5%), 8,377 cases had no military experience (90.5%), one refused to answer the question and one responded “I don't know.” Age ranged from 17 to 85 years. The distribution of ages is plotted in Figure 2. See Table 1 for a breakdown of WARHICS class for left and right ears. Table 2 shows the distribution of answers to the question “Which statement best describes your hearing (without a hearing aid)?”

**Table 1:** Distribution of WARHICS classes in left and right ears.

**WARHICS**	**Frequency**	**Percentage**	**Frequency**	**Percentage**
**class**	**(L)**	**(L)**	**(R)**	**(R)**
1	3,761	40.62%	3,955	42.72%
2	1,754	18.95%	1,711	18.48%
3	1,505	16.26%	1,499	16.19%
4	931	10.06%	822	8.88%
5	760	8.21%	781	8.44%
6	210	2.27%	177	1.91%
7	334	3.61%	313	3.38%
8	3	0.03%	0	0.00%

**Table 2:** Distribution of responses to the question “Which statement best describes your hearing (without a hearing aid)?”

**Response**	**Frequency**	**Percentage**
Excellent	5,027	54.30%
Good	3,103	33.52%
A little trouble	769	8.31%
Moderate hearing trouble	251	2.71%
A lot of trouble	100	1.08%
Deaf	5	0.05%
Refused	0	0.00%
Don't Know	1	0.01%

## Analysis

Three machine learning algorithms were trained on the dataset to predict WARHICS class: random forest (RF), support vector machines with a radial kernel (SVM Radial), and k-nearest neighbors (KNN). Accuracy was used to assess the efficacy of the algorithms.

The 9,256 data points for the left ears were split into two sub-datasets: one for training (80% of the data: 7,407 cases) and one for validation (20% of the data: 1,849 cases). Only three cases were classified as WARHICS class 8, so one of these cases was forced into the validation dataset. The other two cases classified as WARHICS class 8 were used in the training dataset. The right ear data (*N* = 9,256) were used for a second round of validation and testing.

## Results

**Figure 3:**
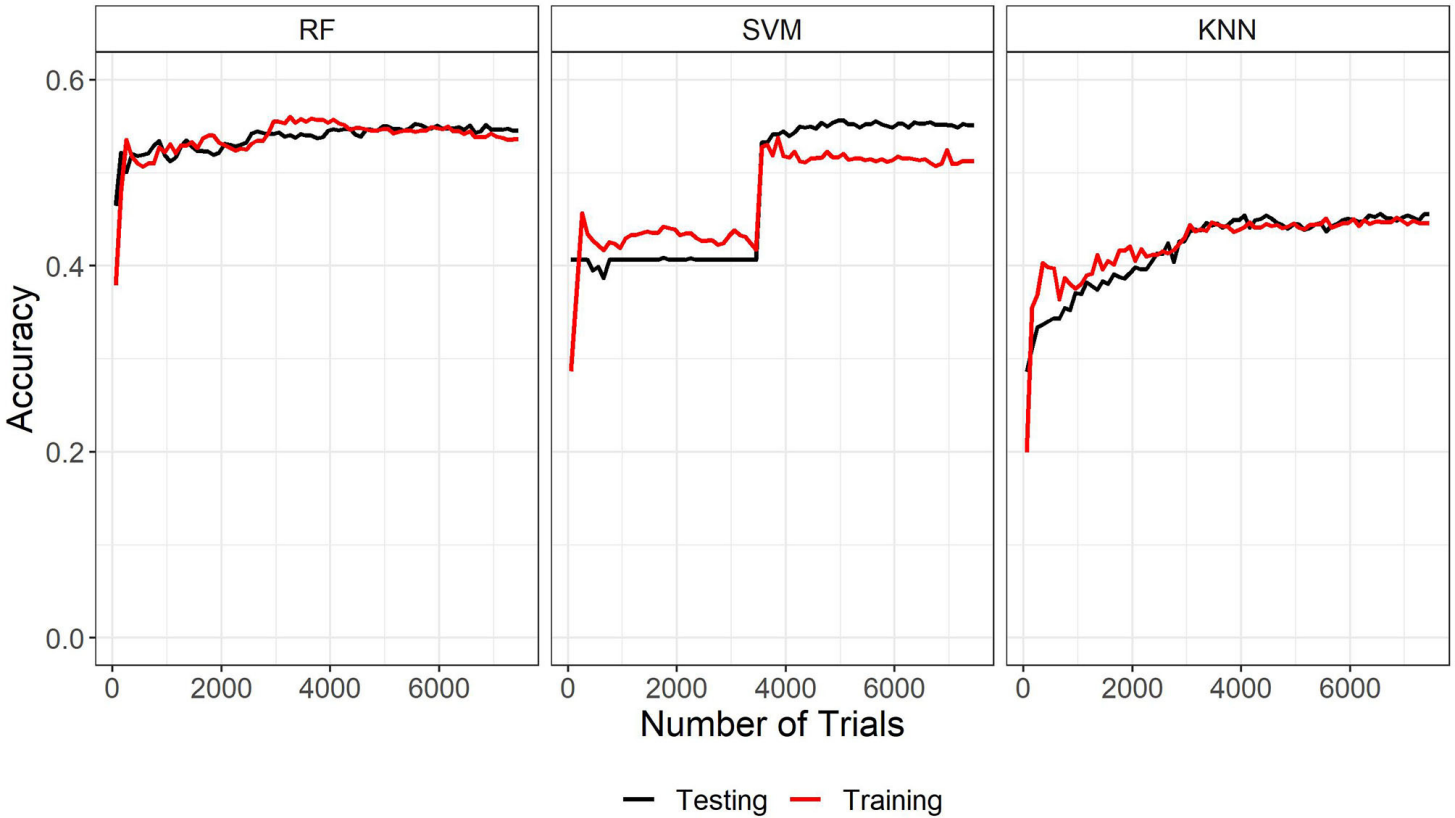
Learning curves of the three machine learning algorithms trained on the left ear data. RF, Random Forest; SVM, Support vector machines with radial kernel; KNN, k-nearest neighbors.

The three machine learning algorithms were assessed based on the time they took to run using a 2.8 GHz 11th Generation Intel® Core™ i7 processor with no parallel processing, the overall accuracy, and learning curves. See Table 3 for run time, accuracy, and final parameters fit. Learning curves for the three algorithms are plotted in Figure 3. WARHICS class 8 was excluded from the learning curves because that class was rare.

**Table 3:** Run time, accuracy, and the final fit parameters for a random forest (RF), support vector machines with a radial kernel (SVM Radial), and k-nearest neighbors.

**Algorithm**	**Run Time**	**Accuracy**	**Parameters**
RF	224.91 s	0.54	mtry = 2
SVM Radial	460.99 s	0.51	sigma = 0.23, C = 1
KNN	3.69 s	0.45	k = 9

*Run time is included here for completeness, but a machine learning algorithm could be implemented for the purposes discussed in this paper using a remote server to bypass the run time*.

The learning curves and accuracy indicate that the random forest algorithm is the best algorithm among the three. Although run time is another typical metric for measuring machine learning algorithms, it is not an important factor here. Run time information is only relevant for assessing the algorithms if they needed to be run each time they had to categorize a new case. In the applications discussed in the present study, this would not be the case because the chosen algorithm could be implemented on a remote server and called when needed. The run time is reported here for completeness. The learning curves show a normal pattern of results and a good fit for both the RF and KNN algorithms. The large jump in performance around 3,500 trials and the wide gap in performance at the end of the training indicate that the SVM Radial model is not a good fit for these data. Based on run time, accuracy, and learning curves, RF was used to predict WARHICS class.

RF prediction efficacy was assessed using confusion matrices. The left ear validation dataset saw significantly higher accuracy than the no information rate (Acc = 0.5462, NIR = 0.4067, *p* < 0.001). Cohen's kappa was calculated as 0.35 which signifies a fair agreement between the reference and prediction (16). The confusion matrix from which these values were calculated is shown in Table 4 along with within-class precision and recall calculated following the guidelines laid out in Sokolova and Lapalme (17). Overall, the model performs best at classifying individuals with no clinical hearing loss (WARHICS class 1). The algorithm performs less well at identifying individuals that fall into WARHICS class 2 and WARHICS class 5.

**Table 4:** Confusion matrix results of left ear machine learning predictions.

		**Reference**								
		**1**	**2**	**3**	**4**	**5**	**6**	**7**	**8**	**Precision**
Prediction	1	**705**	244	92	13	31	2	5	0	0.65
	2	11	**13**	8	2	1	0	1	0	0.36
	3	36	92	**168**	72	55	2	14	0	0.38
	4	0	0	26	**89**	40	19	10	0	0.48
	5	0	0	5	8	**11**	10	12	0	0.24
	6	0	0	0	1	5	**5**	2	0	0.38
	7	0	1	1	1	9	4	**22**	1	0.56
	8	0	0	0	0	0	0	0	**0**	0
Recall		0.9	0	0.6	0.5	0.07	0.12	0.3	0	

*Columns represent the reference WARHICS classes from the NHANES dataset. Rows represent the predicted WARHICS classes from the RF algorithm. Numbers along the diagonal (in bold) count the number of correct classifications. Numbers above the diagonal count the number of classifications where the predicted WARHICS class is less severe than the reference WARHICS class (underprediction of loss). Numbers below the diagonal count the number of classifications where the predicted WARHICS class is more severe than the reference WARHICS classification (overprediction of loss). Overall, the machine learning algorithm correctly predicted 54.79% of losses, underpredicted 34.40% of losses, and overpredicted the remaining 10.82% of losses. Within-class precision and recall for these data are presented in the margins. The precision and recall values for WARHICS class 8 are anomolous because there was only 1 case in the validation dataset and it was misclassified. They are included here for completeness*.

Right ear data were used to test the RF algorithm. The same model trained on 80% of the left ear data was used to predict the classification for all 9,258 cases of right ear data. Again, accuracy was significantly greater than the no information rate (Acc = 0.5583, NIR = 0.4273, *p* < 0.001). Cohen's kappa showed fair agreement between the machine learning algorithm and the reference classifications (Cohen's kappa = 0.3573). The confusion matrix from which these values were calculated is shown in Table 5 along with within-class precision and recall. The algorithm shows a similar pattern of results for the right ear data as it did for the left ear validation dataset, though the algorithm seems to have more success classifying listeners in WARHICS class 5 for the right ears than it did for the left.

**Table 5:** Confusion matrix results of right ear machine learning predictions.

		**Reference**								
		**1**	**2**	**3**	**4**	**5**	**6**	**7**	**8**	**Precision**
Prediction	1	**3,712**	1,185	482	73	140	0	34	0	0.66
	2	46	**56**	59	13	6	0	3	0	0.31
	3	195	455	**803**	300	291	8	75	0	0.38
	4	1	10	139	**374**	197	92	39	0	0.44
	5	0	2	5	45	**97**	37	41	0	0.43
	6	0	0	0	5	14	**25**	16	0	0.42
	7	1	3	9	12	36	15	**105**	0	0.58
	8	0	0	0	0	0	0	0	**0**	0
Recall		0.94	0.03	0.5	0.5	0.12	0.14	0.34	0	

*This table is laid out the same way as Table 4. Overall, the machine learning algorithm correctly predicted 55.88% of losses, underpredicted 33.39% of losses, and overpredicted the remaining 10.73% of losses. Within-class precision and recall for these data are presented in the margins*.

## Discussion

There are several ways to assess the real-world efficacy of a machine learning algorithm. At the end of the day, we want to know how accurate the algorithm is; however, “accuracy” can be conceived of in different ways. We will explore two rules for assessing accuracy: a strict rule, and a practical rule.

The strict rule states that *any* mismatch between the predicted WARHICS class and the reference WARHICS class is a miss. For example, if the listener has a reference WARHICS class of 5 and they are categorized as WARHICS 5, this is a hit. If they are categorized as WARHICS 4, this is a miss. The practical rule is based on the difference in expected speech audibility due to a mismatch between the predicted and reference WARHICS classes. For details on calculation of this this rule, please see the Supplementary Materials.

By the strict rule, the machine learning algorithm correctly categorizes a loss roughly 55% of the time—about 1 in 2 individuals. This is certainly below the desired success rate, but this is due to the intentional lack of information provided to the machine learning algorithm. If all pure tone frequencies were included, the machine learning algorithm would have been significantly more accurate; however, this was not the goal of the present study. The intent was to see how accurately the machine learning algorithm could predict audiometric configurations given *limited* information that one might expect to have when using uncalibrated equipment in a person's home, similar to the approach suggested by Van Tasell. A different machine learning approach achieved around 90% accuracy across different audiometric configurations using judgments provided by three licensed audiologists about the configuration, severity, and symmetry of participant's losses (18). However, such an approach requires more resources and is subject to variability according to the experts being consulted. An advantage of the method tested here, despite its lower accuracy by the strict rule, is its ability to be fully automated and implemented in remotely-conducted auditory experiments where expert judgment cannot be easily applied.

Given these results, the practical rule may be the appropriate way to describe the results of the present experiment if the machine learning solution presented here were used to predict thresholds for a speech intelligibility experiment. By the practical rule, the machine learning algorithm succeeds 88.3% of the time. This success rate is much better than the strict rule partly due in part to a laxer criterion for counting a success. However, the practical rule does as its name implies: uses a practical threshold for success based on the audibility that would be achieved for presented speech stimuli. Eighty eight point three percent of the cases would still be predicted to score 95% correct on sentence materials, even when underamplified. If a machine learning solution were used in this context, a researcher may be able to identify whether a listener received the correct gain or not. A researcher might be able to identify which of the remaining 11.7% were misclassified by looking at volume control (presumably, listeners that were overamplified would turn the volume down to a comfortable level), or by devising a threshold test at the outset of the experiment to identify those that were underamplified. Such methods are speculative here and would need to be refined further in the future.

User-operated tests could be applied inside and outside of the clinic. In the clinic, it could be used to improve efficiency. An audiologist that needs to only measure two or three air conduction thresholds in conjunction with a short questionnaire would save a substantial amount of time, improving the efficiency of clinic operations. The saved time could then be used for other diagnostic tests or counseling. This is consistent with calls to action for audiologists to focus on more sophisticated measures, expert interpretation, and patient counseling, vs. spending a majority of their appointment time manually adjusting the levels produced by a pure-tone audiometer (19, 20). With regard to in-home testing, measurement of audiometric thresholds is becoming a reality with devices like the AMTAS Home Hearing Test (4, 5). Such in-home devices are expensive and must be physically provided to the patient if it is important that the test be calibrated to provide accurate results. However, if a patient were provided with an online link via their home computer, a first fit could be estimated with only two pure tone thresholds, a short questionnaire, and without the need for precisely calibrated presentation levels. If a method was able to accurately predict an individual's WARHICS class, a hearing aid might then be provided with the initial frequency-gain response set according to the predicted audiogram and with a margin of adjustment considered acceptable for the user. The margin of adjustment would likely cover the range of the WARHICS class assigned to the patient. Such a range would acknowledge the fact that the machine learning solution presented here does not predict a specific audiogram, but rather a range of possible audiograms. Setting a range of adjustment values could be a potential solution to this problem. Support for this method comes from a recent paper suggesting that hearing aids set by the user using a smartphone app can provide outcomes that are as good as—or better than—those provided by the traditional audiologic best practices (21). Using machine learning to restrict the adjustment range could speed up the process of self-fitting for the patient. A combination of user-adjusted response and response constraints based on predicted audiogram would guard against situations where the user chooses a response that is inadequate or inappropriate for their hearing loss.

As a caution, if in-home testing becomes a broadly accepted option in the future, careful steps will need to be taken to make sure that patients have a pathway for follow-up likely including a full audiogram, medical care, and that treatable audiologic disorders are not missed. One questionnaire, the Consumer Ear Disease Risk Assessment (CEDRA), effectively screens for serious audiologic disorders (22). CEDRA or a similar questionnaire could be used as a supplement during at-home hearing screening. In view of data that perceived hearing disability is not strongly related to pure-tone thresholds [e.g., (23)], additional information may be needed to guide provision of amplification once a hearing loss has been identified. Nonetheless, recent developments in auditory science (accelerated by effects of COVID-19 on elective medical care) suggest that remote, at-home or other user-centered assessment techniques will play a role in future treatment options. That said, the present machine learning algorithm is not ready for deployment on a massive scale in clinical settings. The solution presented here would need to be fine-tuned, validated, and likely included in a battery of other tests.

For research studies, a researcher using the approach described here might be able to administer in-home speech tests to individuals with hearing loss without needing detailed knowledge of the participant's computer, headphones, or sound card. Although environmental factors (e.g., road noise, background voices, construction, pets, etc.) cannot be controlled for using this method, the experimenter can coarsely estimate the class of a participant's loss and apply the appropriate gain. In view of the known difficulties in accurately predicting loudness perception from pure-tone thresholds (24), it would be prudent of that experimenter to include a restricted volume adjustment for the participant (perhaps one that maintains an acceptable SII, as described above) in the case of loudness discomfort. Such an approach would benefit the field of hearing research by greatly expanding the sample size and sample demographics without incurring much extra cost.

## Data Availability Statement

Publicly available datasets were analyzed in this study. This data can be found here: https://wwwn.cdc.gov/nchs/nhanes/default.aspx National Health and Nutrition Examination Survey (NHANES) years 1999-2012 and 2015-2016.

## Author Contributions

GE: responsible for data processing, analysis, figures, tables, and Discussion. PS: responsible for Introduction, Discussion, framing, and editing. Both authors contributed to the article and approved the submitted version.

## Conflict of Interest

The authors declare that the research was conducted in the absence of any commercial or financial relationships that could be construed as a potential conflict of interest.

## Publisher's Note

All claims expressed in this article are solely those of the authors and do not necessarily represent those of their affiliated organizations, or those of the publisher, the editors and the reviewers. Any product that may be evaluated in this article, or claim that may be made by its manufacturer, is not guaranteed or endorsed by the publisher.
