# Supplementary material for: Using Machine Learning and the National Health and Nutrition Examination Survey to Classify Individuals With Hearing Loss
Source: Front Digit Health. 2021 Aug 18;3:723533. doi: 10.3389/fdgth.2021.723533 (PMC8521948; doi:10.3389/fdgth.2021.723533)
Supplement: Supplementary file 1 [file Data_Sheet_1.docx]

# Supplemental Materials: Calculating the Practical Rule

In order to calculate the practical rule, we modeled a hypothetical situation in which a predicted WARHICS class might be used to prescribe hearing aid gain (either for an individual hearing aid, such as a direct-to-consumer device, or as part of an auditory experiment). In this hypothetical situation, the participant receives gain following the NAL-NL2 gain rule (Keidser, Dillon, Flax, Ching, & Brewer, 2011) for the most severe loss consistent with their predicted WARHICS class. If a participant is predicted to fall in WARHICS class 2, but the reference is WARHICS class 3, this participant will be underamplified relative to the gain they would have received if their audiogram was known.

The degree to which a mismatch would affect their ability to understand speech can be estimated using the Speech Intelligibility Index (SII; ANSI, 1997). The inputs to the SII were the most extreme predicted WARHICS class thresholds minus the gain prescribed following the NAL-NL2 gain rule. For example, if a listener is predicted to fall in WARHICS class 2 (most extreme thresholds are 25, 25, 25, 30, and 30 dB HL at 0.5, 1, 2, 4, and 8 kHz) the appropriate gain for that listener would be 0, 0, 7.3, 18.1, and 2.8 dB SPL at 0.5, 1, 2, 4, and 8 kHz, respectively. If that person’s true class was WARHICS class 3 (most extreme thresholds are 25, 25, 25, 45, and 65 dB HL at 0.5, 1, 2, 4, and 8 kHz), the prescribed gain combined with their thresholds would produce effective thresholds of 25, 25, 17.7, 26.9, and 62.2 dB HL. The effective thresholds were used as the input to the SII. The SII was calculated using a conversational-level input (62.35 dB SPL) in a relatively quiet environment (10 dB SNR). The SII standard states that any communication system with an SII value below 0.45 constitutes a poor communication system (ANSI, 1997). In addition, any SII value can be used to predict percent correct on a speech intelligibility task using a transfer function. Based on previous research, an SII of 0.45 should produce approximately 95% correct speech intelligibility for sentences (Ching, Dillon, & Byrne, 1998; Kryter, 1962; Magnusson, 1996). Given that 95% correct speech recognition for sentences is extremely good performance, the practical rule will consider any SII values falling below 0.45 to be failures. For the purpose of this interpretation, any SII values falling above or equal to 0.45 will be considered successes. This concept is illustrated by the values in Table S1.

In any speech task, precautions must be taken to ensure that presented stimuli are safe and comfortable for listeners during experimental or clinical procedures. Following from the SII values, it’s clear that underamplification can lead to inadequate audibility and resulting problems completing the task. It is also important to consider the consequences of overamplifying the listener. Overamplification can lead to loudness discomfort or in some cases could even result in hearing damage. This is critical if the task is administered under remote test conditions where the listener is not monitored. Experimenters or clinicians will need to devise ways to address this. One potential solution, and a necessary precaution in the authors’ view, is to give the listener some degree of control over output level. The degree to which this compromises the results for different tasks will vary, but it can be easily calculated for speech intelligibility tasks using the Speech Intelligibility Index.

In order to address concerns about overamplification, the practical rule will also consider overamplification by 10 dB within any individual frequency a failure. Ten dB is used since that is the maximum adjustment which would generally be considered an acceptable allowed range for a listener adjusting their own hearing aid volume control (Cox & Alexander, 1991; Mueller, Hornsby, & Weber, 2008). As an example, a participant predicted to fall into WARHICS class 5 but whose reference class is 3 would receive 10.1 dB more gain than necessary at 1 kHz. Regardless of the amount of mismatch at any other frequency, this case would be considered a failure. It should be noted that cases where the practical rule failed due to overamplification are extremely rare, occurring in only 1.19% of left ear validation cases and 0.95% of right ear validation cases; however, overamplification is important to guard against and is emphasized here for that reason.

Table S1. SII values calculated based on receiving NAL-NL2 gains for the predicted WARHICS class when a participant’s true WARHICS class is the reference. The diagonal shows SII values for receiving the appropriate amplification. Above the diagonal, participants would be underamplified. The SII was calculated for a normal speaking voice (62.35 dB) in a relatively quiet environment (10 dB SNR). Values that fail the practical rule (SII < 0.45 or overamplification in any octave frequency by more than 10 dB) are shaded red. An SII value of 0.45 corresponds to approximately 95% correct speech intelligibility for sentence materials (Ching, et al., 1998; Kryter, 1962; Magnusson, 1996).

|  |  | Reference | | | | | | |  |
| --- | --- | --- | --- | --- | --- | --- | --- | --- | --- |
|  |  | 1 | 2 | 3 | 4 | 5 | 6 | 7 | 8 |
| Prediction | 1 | **0.72** | 0.71 | 0.68 | 0.49 | 0.28 | 0.24 | 0.00 | 0.00 |
|  | 2 | 0.72 | **0.71** | 0.71 | 0.51 | 0.30 | 0.24 | 0.02 | 0.00 |
|  | 3 | 0.73 | 0.72 | **0.71** | 0.55 | 0.31 | 0.25 | 0.06 | 0.00 |
|  | 4 | 0.75 | 0.73 | 0.71 | **0.66** | 0.38 | 0.26 | 0.12 | 0.00 |
|  | 5 | 0.75 | 0.73 | 0.71 | 0.67 | **0.52** | 0.39 | 0.13 | 0.00 |
|  | 6 | 0.75 | 0.74 | 0.71 | 0.68 | 0.59 | **0.47** | 0.14 | 0.00 |
|  | 7 | 0.75 | 0.75 | 0.71 | 0.70 | 0.62 | 0.56 | **0.21** | 0.00 |
|  | 8 | 0.75 | 0.75 | 0.73 | 0.71 | 0.71 | 0.63 | 0.47 | **0.09** |

**REFERENCES**

Mueller HG, Hornsby BW, Weber JE. Using trainable hearing aids to examine real-world preferred gain. *J Am Acad Audiol*. (2008) 19:758–73. doi: 10.3766/jaaa.19.10.4

Keidser G, Dillon H, Flax M, Ching T, Brewer S. The NAL-NL2 prescription procedure. *Audiol Res*. (2011) 1:e24. doi: 10.4081/audiores.2011.e24

Cox RM, Alexander GC. Preferred hearing aid gain in everday environments. *Ear Hear.* (1991) 12:123–6. doi: 10.1097/00003446-199104000-00008

Ching TYC, Dillon H, Byrne D. Speech recognition of hearing-impaired listeners: predictions from audibility and the limited role of high-frequency amplification. *J Acoust Soc Am*. (1998) 103:1128–40. doi: 10.1121/1.421224

Kryter KD. Methods for the calculation and use of the articulation index. *J Acoust Soc Am*. (1962) 34:1689–97. doi: 10.1121/1.1909094

ANSI A. S3. 5-1997 (R2017). *Methods for Calculation of the Speech Intelligibility Index.* American National Standards Institute (1997).

Magnusson L. Speech intelligibility index transfer functions and speech spectra for two Swedish speech recognition tests. *Scandinavian audiology*. (1996) 25:59–67. doi: 10.3109/01050399609047557
